# Electrocardiographic P-Wave Indices in Metabolic Dysfunction-Associated Fatty Liver Disease and Their Relationship to Hepatic Fibrosis Risk

**DOI:** 10.3390/jcm14134650

**Published:** 2025-07-01

**Authors:** Muhammet Salih Ateş, Erdoğan Sökmen

**Affiliations:** Department of Cardiology, Kırşehir Ahi Evran Training and Research Hospital, Kırşehir 40100, Turkey; muhammet.ates@ahievran.edu.tr

**Keywords:** MAFLD, P-wave peak time, P-wave dispersion, ECG, fibrosis detection

## Abstract

**Background/Objectives**: Metabolic dysfunction-associated fatty liver disease (MAFLD) is linked to cardiovascular complications, including atrial fibrillation. P-wave indices (PWIs) reflect atrial conduction heterogeneity but have not been fully evaluated in MAFLD. To compare PWIs in MAFLD patients versus controls, assess their association with fibrosis severity, and evaluate their diagnostic performance for MAFLD and fibrosis. **Methods**: In this retrospective single-center study, 447 subjects were included (noMAFLD: Fatty Liver Index (FLI) < 30 without metabolic dysfunction, n = 205; MAFLD: FLI ≥ 60+ ≥ 1 metabolic risk factor, n = 242). Among MAFLD subjects, the non-alcoholic fatty liver disease (NAFLD) Fibrosis Score (NFS) stratified lower (NFS ≤ −1.455; n = 170), and there was a higher fibrosis risk (NFS > −1.455; n = 72). Standard 12-lead ECGs were digitized for offline PWI measurement. Statistical analyzes included group comparisons, multivariable logistic regression, and ROC curve analysis. **Results**: MAFLD patients exhibited a longer PWPT-D2 (63 ± 12 vs. 52 ± 10 ms, *p* = 0.003), PWPT-V1 (68 ± 14 vs. 60 ± 13 ms, *p* = 0.005), PWdis (55 ± 13 vs. 46 ± 11 ms, *p* = 0.010), and PTFV_1_ (38 [31–46] vs. 28 [22–34] mm·ms, *p* = 0.021) compared with controls. Within MAFLD, a higher fibrosis risk was associated with a further PWI prolongation (all *p* < 0.015). Multivariate analysis identified PWPT-D2 (OR 1.05 per ms; 95% CI 1.02–1.08; *p* = 0.002) and PWDIS (OR 1.03 per ms; 95% CI 1.00–1.06; *p* = 0.048) as independent MAFLD predictors. ROC curves showed PWPT-D2 had the highest AUC for MAFLD detection (0.78; 95% CI 0.72–0.84) and fibrosis (0.82; 95% CI 0.76–0.88). Combining PWPT-D2 with BMI and waist circumference improved MAFLD discrimination (AUC 0.89; 95% CI 0.85–0.93; *p* < 0.001 vs. PWPT-D2 alone). **Conclusions**: PWPT-D2 and PWdis are significantly prolonged in MAFLD and more so with advanced fibrosis. PWPT-D2 may be a simple, noninvasive ECG marker for MAFLD screening and fibrosis staging, particularly when combined with anthropometric measures.

## 1. Introduction

Metabolic dysfunction-associated fatty liver disease (MAFLD) became evident as the most prevalent chronic liver disease all over the world, affecting approximately 25–30% of adults in Western populations and over 30% in Asia [1,2]. MAFLD encompasses a range of liver conditions that progress histologically from uncomplicated fat accumulation in the liver (hepatic steatosis) to more severe stages including metabolic dysfunction-associated steatohepatitis, the development of fibrosis, cirrhosis, and eventually hepatocellular carcinoma [3]. Beyond the progression to advanced liver disease, MAFLD confers a substantial risk for extrahepatic complications, notably cardiovascular disease (CVD), which represents the number one reason for morbidity and mortality in this population [4,5].

Epidemiological investigations have consistently demonstrated that subjects having MAFLD face an elevated risk of atrial fibrillation (AF), with several large cohort analyzes finding a two- to three-fold increase in incident AF compared to non-MAFLD controls [6,7]. Moreover, the prolongation of ventricular repolarization markers—specifically QT and QTc intervals—has been reported in MAFLD, suggesting a greater predisposition to ventricular arrhythmias and sudden cardiac death [8,9]. Other indices of ventricular repolarization such as Tp-e interval and Tp-e/QTc ratio, important parameters rendering the heart susceptible to ventricular tachyarrhythmias, were also demonstrated to show elevations when compared with non-MAFLD controls [10]. However, while these findings implicate both atrial and ventricular electrophysiological disturbances in MAFLD, a comprehensive evaluation of P-wave indices (PWIs)—which specifically reflect atrial conduction heterogeneity and are strong predictors of AF onset—has been lacking in MAFLD cohorts.

PWIs on a standard 12-lead ECG, such as P-wave duration (PWD), P-wave dispersion (PWdis), P-wave peak time in D2 (PWPT-D2), P-wave peak time in V_1_ (PWPT-V1), and P-wave terminal force in V_1_ (PTFV_1_), provide noninvasive markers of atrial remodeling and conduction delay [11,12,13]. Prolonged PWPT and increased PWdis have been independently associated with elevated left atrial (LA) pressures and higher rates of incident AF in various populations, including the subjects with type 2 diabetes mellitus (DM) and hypertension [14,15]. Given that MAFLD is strongly linked to systemic inflammation, insulin resistance, and myocardial fibrosis—mechanisms that promote atrial structural remodeling—it is plausible that PWIs might be altered early in MAFLD patients, even in the absence of clinically manifested AF [16,17].

To date, however, no study has systematically characterized PWIs in MAFLD or assessed their utility for identifying patients at risk for atrial arrhythmias in this setting. Existing investigations have focused primarily on QT/QTc prolongation or on ultrasound-based surrogates of LA size rather than on direct ECG markers of atrial conduction. As a result, the full extent to which PWIs reflect atrial electromechanical remodeling in MAFLD remains unknown.

To address these gaps, our study was designed with three primary objectives: (1) to compare key PWIs between MAFLD patients and non-MAFLD controls; (2) to assess how these indices vary according to fibrosis risk stratified by the non-alcoholic fatty liver disease (NAFLD) Fibrosis Score; and (3) to evaluate the diagnostic performance of PWIs—alone and in combination with anthropometric measures (BMI and waist circumference)—for identifying MAFLD and higher fibrosis. By systematically evaluating PWIs, markers of subclinical atrial electrical remodeling, this work represents the first comprehensive effort to link atrial conduction heterogeneity with underlying hepatic pathology in MAFLD.

## 2. Methods

### 2.1. Study Population

**Figure 1:**
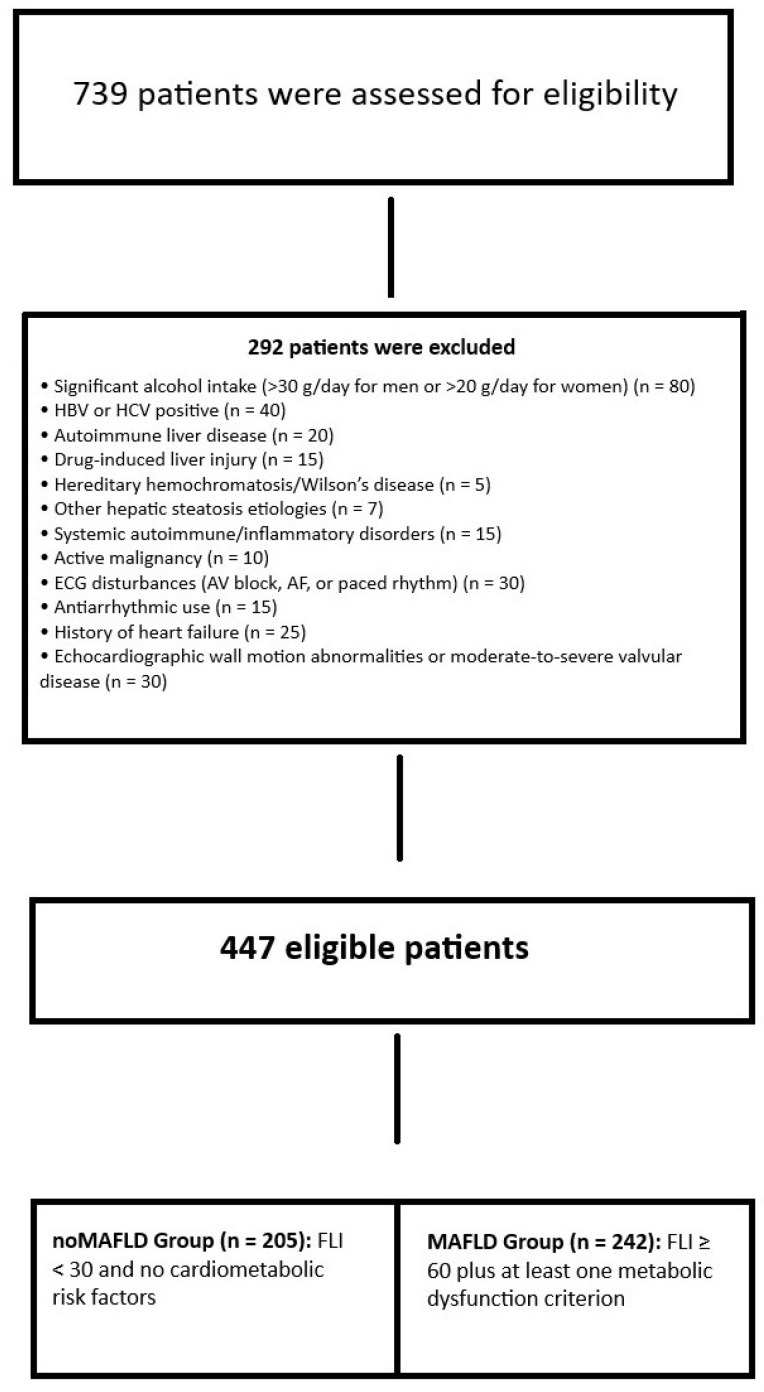
Patient inclusion flowchart.

This study is a retrospective analysis of prospectively collected clinical and electrocardiographic data from adult patients undergoing routine cardiac evaluation. Participants were identified through a retrospective review of all individuals aged ≥18 years who attended cardiology outpatient clinics between May 2022 and May 2024. Demographic and clinical information, along with laboratory results, echocardiographic measurements, and electrocardiographic tracings, were extracted from the institutional electronic health records. Patients were excluded if they had significant alcohol consumption (>30 g/day for men or >20 g/day for women), autoimmune liver disease, positive serology for hepatitis B or C, drug-induced liver injury, hereditary hemochromatosis, Wilson’s disease, or any other known etiology of hepatic steatosis. Additional exclusion criteria comprised systemic autoimmune or inflammatory disorders, active malignancy, ECG evidence of atrioventricular conduction disturbances, atrial fibrillation or paced rhythm, current use of antiarrhythmic medications, a history of heart failure, echocardiographic findings of segmental wall motion abnormalities, or moderate to severe valvular heart disease. A total of 739 participants were evaluated for eligibility, and, after applying these criteria, the remaining cohort composed of 447 eligible participants was stratified by the Fatty Liver Index (FLI): individuals with FLI < 30 and no cardiometabolic risk factors were allocated to the noMAFLD control group, while those with FLI ≥ 60 in conjunction with at least one metabolic dysfunction criterion were assigned to the MAFLD group. Figure 1 represents the patient inclusion flowchart. Our study complied with the declaration of Helsinki, and the local ethics committee approved our study.

#### The Diagnosis of MAFLD and the Evaluation of Hepatic Fibrosis Severity

In accordance with current clinical practice guidelines [18] and after excluding secondary causes of fatty liver, MAFLD was identified using the Fatty Liver Index (FLI) [19]. The FLI integrates four variables—serum triglycerides, body mass index (BMI), gamma-glutamyl transferase (GGT), and waist circumference—via the following equation [19]:

FLI = (0.953 × ln [triglycerides (mg/dL)] + 0.139 × BMI (kg/m^2^) + 0.718 × ln [GGT (U/L)] + 0.053 × waist circumference (cm) − 15.745)

FLI values ≥ 60 were associated with a strong diagnostic capability for ultrasound-detected hepatic steatosis, yielding a sensitivity of 96%, specificity of 92.5%, and an AUC of 0.92 [20]. An FLI score ≥ 60 in conjunction with evidence of metabolic dysfunction was widely accepted as diagnostic of MAFLD [21]. Specifically, metabolic dysfunction is defined by the presence of one of the following three conditions: (1) overweight or obesity (BMI ≥25 kg/m^2^); (2) type 2 DM; or (3) at least two of the following risk factors: waist circumference >94 cm in men or >80 cm in women; triglycerides >150 mg/dL; HDL-cholesterol <40 mg/dL in men or <50 mg/dL in women; fasting glucose >100 mg/dL; or blood pressure ≥130/85 mmHg [21].

For patients meeting MAFLD criteria, a noninvasive assessment of hepatic fibrosis was performed using the NAFLD Fibrosis Score (NFS) [22]. The NFS incorporates age, BMI, the presence of type 2 DM, the aspartate aminotransferase to alanine aminotransferase ratio, platelet count, and serum albumin according to the following formula [22]:

NFS = −1.675 + 0.037 × Age (years) + 0.094 × BMI (kg/m²) + 1.13 × (DM: 1 if present, 0 if absent) + 0.95 × [AST/ALT ratio] − 0.013 × Platelet count (10⁹/L) − 0.66 × Albumin (g/dL)

Based on original validation study, an NFS ≥ 0.675 indicates advanced fibrosis, while an NFS ≤ −1.455 effectively rules it out; scores between −1.455 and 0.675 are considered indeterminate and warrant further evaluation [23]. We dichotomized our MAFLD group into two subgroups as those with NFS ≤ −1.455 (lower fibrosis risk group) and those with NFS > −1.455 (higher fibrosis risk group).

With the patient in a standing position, waist circumference was measured at the midpoint between the lower rib margin and the iliac crest. BMI was computed as weight (kg) divided by height squared (m^2^). Laboratory results were obtained from fasting blood samples.

### 2.2. Electrocardiographic Analysis

Electrocardiographic recordings were obtained using a conventional 12-lead resting ECG device (MAC 2000, GE Medical Systems, Milwaukee, WI, USA), with settings of 25 mm/s paper speed and 10 mm/mV calibration. All tracings were digitized and imported into Adobe Photoshop CC (Adobe Systems Inc., San Jose, CA, USA) for offline analysis. Two cardiologists (F.K. and A.Y.), each blinded to patient clinical status and echocardiographic findings, independently measured atrial and ventricular parameters. For atrial conduction assessment, P-wave onset and offset were identified in derivation 2 to determine PWD, and PWmax and PWmin durations were similarly measured across all 12 leads to calculate PWdis. The P-wave peak time was accepted as the time period between the P-wave onset to the point of maximal positive deflection in derivation 2 (PWPT-D2) and V1 (PWPT-V1). PTFV1 was quantified as the area under the terminal negative deflection (measured in mm·ms) on the V1 tracing. All PWIs were obtained from three consecutive sinus beats, with the mean value recorded. Ventricular repolarization intervals (QT and QTc) were measured in derivation 2 as the interval from the onset of QRS to the T-wave end, with QTc calculated using Bazett’s formula (QTc = QT/√RR).

Inter- and intra-observer reproducibility was assessed in 30 randomly selected ECGs. Intra-observer coefficients of variation (CV) for PWD, PWdis, PWPT-D2, PWPT-V1, and PTFV1 were 3.2%, 3.8%, 4.1%, 4.3%, and 4.0%, respectively. Inter-observer CVs for the same parameters were 5.6%, 6.1%, 6.5%, 6.8%, and 6.2%, respectively. Ventricular measurements yielded intra-observer CVs of 2.5% (QT) and 2.8% (QTc), with corresponding inter-observer CVs of 4.5% and 4.9%. Discrepancies >10 ms for any interval prompted re-measurement and consensus between the two readers.

### 2.3. Echocardiography

Echocardiographic examinations were performed in all subjects using Vivid S5 (GE Vingmed, Horten, Norway). Standard parasternal long- and short-axis views, along with apical four- and two-chamber perspectives, were used to acquire two-dimensional and M-mode echocardiographic images. Left ventricular ejection fraction (LVEF) was calculated using the biplane Simpson’s method based on the apical views. LA area was traced at end-systole in the apical four-chamber view, excluding pulmonary venous confluence and LA appendage, and the mean of three consecutive beats was recorded. Left ventricular diastolic function was assessed by a pulsed-wave Doppler interrogation of transmitral inflow (peak E and A velocities, E/A ratio, and deceleration time) and by tissue Doppler imaging (TDI) of the mitral annulus. Medial and lateral annular early diastolic velocities (e′) were measured by TDI in the apical four-chamber view, and the E/e′ ratios were calculated separately using medial and lateral e′ velocities. M-mode-derived measurements of left ventricular end-diastolic dimension, interventricular septal thickness, and posterior wall thickness were obtained from the parasternal long-axis window at the level of the mitral valve leaflet tips, with values averaged over three cardiac cycles. All measurements were performed offline by one experienced echocardiographer blinded to clinical and laboratory data.

### 2.4. Statistical Analysis

The statistical analyzes of the current study were fulfilled using IBM SPSS Statistics version 28. The Shapiro–Wilk test was implemented in order to check the continuous variables for normality. Those that were approximately normally distributed were compared with independent samples t-tests; variables that deviated from normality were compared using the Mann–Whitney U test. Categorical variables were compared via Chi-square tests, or the Fisher’s exact test when any expected cell count fell below five. A two-tailed *p* < 0.05 was considered statistically significant.

Next, we evaluated the independent association between each PWIs and FLI using multiple linear regression. In univariate models, each PWI was entered alone with FLI as the dependent variable. In the fully adjusted (multivariate) model, all five PWI were entered simultaneously along with the following covariates: gender, age, BMI, waist circumference, triglycerides, GGT, smoking history, hypertension, DM, and echocardiographic LA area, and lateral E/e′ ratio. The Enter method was used for all predictors. Collinearity was assessed via tolerance (>0.20) and variance inflation factor (VIF < 2.5). Model fit was judged by R^2^, adjusted R^2^, and the overall F-statistic; residual independence was checked using the Durbin–Watson statistic (ideal ≈ 2.0); homoscedasticity and the normality of residuals were confirmed by plotting standardized residuals versus predicted values and inspecting P–P plots. Any case with standardized residual > |3| was examined as a potential outlier but none were excluded.

We implemented a multivariable logistic regression analysis to examine any probable association between continuous PWI and MAFLD status. The analysis followed a hierarchical approach with three sequential models to systematically evaluate these relationships while controlling for confounding variables. Model 1 included baseline adjustments for demographic factors (sex and age) and obesity parameters (BMI and waist circumference). Model 2 added to Model 1 the metabolic covariates (hypertension, DM, smoking history, triglyceride levels, and GGT). Model 3 further incorporated into Model 2 the echocardiographic markers of cardiac remodeling (LA area and lateral E/e′ ratio). All PWI were entered as continuous predictors without dichotomization to maximize statistical power. Continuous variables were standardized prior to analysis using z-score transformation. We assessed model fit through several metrics: the Omnibus test of model coefficients for overall significance, Nagelkerke R^2^ for explained variance, and the Hosmer–Lemeshow test for goodness-of-fit. Multicollinearity was evaluated using variance inflation factors (all < 2.0 in final models), and casewise diagnostics identified no influential outliers (all Cook’s distances < 1). The classification table and area under the ROC curve (AUC) quantified predictive accuracy. All models used the Enter method for variable inclusion and were checked for linearity in the logit using the Box–Tidwell procedure. The hierarchical logistic regression models demonstrated excellent fit, with significant improvement at each step (Omnibus * *p* * < 0.001). The final model (Model 3) explained 41% of MAFLD variance (Nagelkerke R^2^) and showed strong discrimination (AUC 0.83, 95% CI 0.79–0.87). All models were well calibrated (Hosmer–Lemeshow * *p* * > 0.40), with no evidence of multicollinearity (VIFs < 2.0) or influential outliers. Model fit was further evaluated with the Akaike Information Criterion (AIC) and Bayesian Information Criterion (BIC), with smaller values representing a better goodness-of-fit while accounting for model complexity. Classification accuracy progressively improved from 72.3% in Model 1 to 77.6% in Model 3, reflecting an enhanced case identification.

ROC analysis was implemented in order to determine the predictive capability of the individual PWI for MAFLD identification as well as the higher fibrosis subgroup identification among the MAFLD group. A further combined predictive capacity of PWPT-D2, BMI, and waist circumference for MAFLD identification was assessed. Our combined model employed binary logistic regression with MAFLD diagnosis (coded as 0 for controls and 1 for cases) as the dependent variable, incorporating PWPT_D2 using the cutoff of >58 ms obtained from our individual ROC analysis, BMI with the established WHO (World Health Organization) threshold of ≥25 kg/m^2^ indicating overweight/obesity status, and waist circumference applying gender-specific IDF (International Diabetes Federation) metabolic syndrome criteria (≥94 cm for men and ≥80 cm for women). The predicted probabilities generated from the multivariate model were subsequently included into ROC curve analysis, which provided the combined AUC with 95% confidence intervals. We statistically compared this integrated model’s performance against individual predictors through DeLong’s test for correlated ROC curves. To ensure methodological rigor, the analysis included ten-fold cross-validation with AUC recalculation across validation subsets, while all continuous variables were standardized using z-score transformation to enable a direct comparison of effect magnitudes. All ROC analyzes used a two-tailed α = 0.05.

## 3. Results

In final cohort, 447 participants were enrolled as follows: 205 controls without MAFLD (noMAFLD) and 242 patients with MAFLD. Baseline characteristics of the whole cohort is presented in Table 1. There was no significant difference in the mean age (56.8 ± 10.9 vs. 55.5 ± 10.8 years; *p* = 0.180) or sex distribution (47.8% vs. 45.5% female; *p* = 0.622) between the noMAFLD and MAFLD groups. However, MAFLD patients exhibited a significantly higher BMI (29.8 ± 4.7 vs. 24.6 ± 3.2 kg/m^2^; *p* < 0.001) and waist circumference (111.8 ± 12.0 vs. 91.1 ± 6.4 cm; *p* < 0.001). DM (31.0% vs. 12.2%; *p* < 0.001), and hypertension (35.1% vs. 24.9%; *p* = 0.018) prevalences were also greater in the MAFLD group. There were no significant differences in smoking status (21.5% vs. 18.6%; *p* = 0.450) or chronic alcohol use (8.8% vs. 10.3%; *p* = 0.572).

Laboratory data revealed that MAFLD patients had a higher median AST (25 [14–82] vs. 18 [9–45] U/L; *p* < 0.001) and median ALT (26 [13–152] vs. 19 [7–95] U/L; *p* < 0.001). GGT was significantly elevated in MAFLD (36 [11–92] vs. 24 [9–60] U/L; *p* = 0.010), and triglycerides were higher (180.5 ± 100.8 vs. 154.1 ± 70.5 mg/dL; *p* = 0.004). Fasting glucose, total cholesterol, LDL, HDL, bilirubin, ALP, LDH, creatinine, GFR, total protein, albumin, electrolytes, and complete blood count did not differ significantly between groups. The use of oral antidiabetics, insulin, statins, beta blockers, calcium channel blockers, and ACEI/ARBs did not differ significantly.

Echocardiographic parameters demonstrated that MAFLD patients had a significantly larger median LA area (28.3 [24.2–31.5] vs. 22.8 [18.9–25.7] cm^2^; *p* < 0.001) and higher mean E/e′ ratios both laterally (10.5 ± 3.3 vs. 8.2 ± 2.1; *p* < 0.001) and medially (8.9 ± 2.7 vs. 6.5 ± 1.8; *p* < 0.001). The LVEF, E/A ratio, deceleration time, LVEDD, IVS thickness, and PWT were similar between groups.

Among the 242 MAFLD patients, 170 were classified as possessing a lower risk of fibrosis and 72 as a higher risk of fibrosis, and Table 2 demonstrates the comparison between these two subgroups. The median NFS differed significantly (−1.9 [−2.4–1.5] vs. 0.4 [−0.8–1.6]; *p* < 0.001). The MAFLD patients in the higher fibrosis risk group were older (59.7 ± 9.8 vs. 53.2 ± 10.4 years; *p* < 0.001) and possessed a greater BMI (31.5 ± 4.9 vs. 29.1 ± 4.5 kg/m^2^; *p* = 0.002) and waist circumference (115.3 ± 12.8 vs. 110.2 ± 11.5 cm; *p* = 0.013). DM (41.7% vs. 26.5%; *p* = 0.018) and hypertension (44.4% vs. 31.2%; *p* = 0.049) prevalences were greater in the higher fibrosis risk group. There was no difference in sex distribution, tobacco use, and chronic alcohol use.

Laboratory values reflected more severe liver injury among higher fibrosis risk patients: median AST (42 [28–68] vs. 23 [16–38] U/L; *p* < 0.001), median ALT (48 [32–130] vs. 28 [18–44] U/L; *p* < 0.001), and median GGT (58 [35–115] vs. 32 [18–76] U/L; *p* < 0.001) were all higher. Fasting glucose (108 [85–210] vs. 98 [75–182] mg/dL; *p* = 0.005) was elevated in the higher risk group. Triglycerides were higher (193.8 ± 112.4 vs. 175.3 ± 95.2 mg/dL; *p* = 0.032), while HDL, LDL, and total cholesterol did not differ. CRP was increased (5.8 ± 7.3 vs. 4.2 ± 5.1 mg/dL; *p* = 0.048), and platelet count was lower (235.8 ± 68.9 vs. 255.1 ± 72.3 × 103/µL; *p* = 0.046). Fasting glucose, GFR, bilirubin levels, ALP, INR, and other routine labs were comparable.

Echocardiographic data showed that higher fibrosis risk patients had worse diastolic function (E/e′ lateral: 12.2 ± 3.5 vs. 9.5 ± 2.6; *p* < 0.001; E/e′ medial: 9.6 ± 3.0 vs. 7.8 ± 2.3; *p* < 0.001) and a larger LA area (30.5 [26.8–34.0] vs. 27.1 [23.5–30.0] cm^2^; *p* < 0.001). LVEDD was slightly increased (49.8 ± 4.5 vs. 48.1 ± 4.0 mm; *p* = 0.008), as were IVS thickness (10.2 ± 1.5 vs. 9.8 ± 1.3 mm; *p* = 0.041) and PWT (9.7 ± 1.4 vs. 9.2 ± 1.2 mm; *p* = 0.009). LVEF did not differ significantly. Regarding medications, insulin use was significantly higher in the high-risk group (22.2% vs. 5.9%; *p* < 0.001), while oral antidiabetic, statin, beta blocker, and ACEI/ARB use did not differ.

A comparison of the electrocardiographic parameters between noMAFLD and MAFLD groups is presented in Table 3. There was no significant difference in heart rate. Among atrial conduction indices, PWD (94 ± 12 vs. 92 ± 13 ms; *p* = 0.245), PWmax (121 ± 14 vs. 118 ± 15 ms; *p* = 0.189), and PWmin (74 ± 10 vs. 72 ± 9 ms; *p* = 0.157) were similar. In contrast, PWdis was significantly prolonged in MAFLD patients (55 ± 13 vs. 46 ± 11 ms; *p* = 0.010). The median PTFV1 was higher among MAFLD (38 [31–46] vs. 28 [22–34] mm·ms; *p* = 0.021). PWPT-D2 (63 ± 12 vs. 52 ± 10 ms; *p* = 0.003) and in V1 (68 ± 14 vs. 60 ± 13 ms; *p* = 0.005) were both significantly prolonged in MAFLD.

Ventricular parameters showed no significant difference in QRS duration. However, the QT interval was longer in MAFLD (400 ± 36 vs. 380 ± 32 ms; *p* < 0.001), as was QTc (430 ± 30 vs. 410 ± 25 ms; *p* < 0.001). The PR interval and P-wave morphology in V1 were similar between groups.

Within the MAFLD cohort, comparisons between lower and higher fibrosis risk subgroups are detailed in Table 4. The heart rate (77 ± 15 vs. 74 ± 13 bpm; *p* = 0.152) and RR interval (780 ± 151 vs. 811 ± 148 ms; *p* = 0.138) were not significantly different. PWD (95 ± 13 vs. 92 ± 12 ms; *p* = 0.201), PWmax (122 ± 15 vs. 119 ± 14 ms; *p* = 0.183), and PWmin (75 ± 10 vs. 73 ± 9 ms; *p* = 0.241) did not differ by fibrosis status. By contrast, PWdis was longer in the higher risk group (58 ± 14 vs. 52 ± 12 ms; *p* = 0.006). The median PTFV1 was higher in those with a higher fibrosis risk (42 [35–50] vs. 35 [29–42] mm·ms; p = 0.015). PWPT-D2 (66 ± 13 vs. 60 ± 11 ms; *p* = 0.008) and PWPT-V1 (71 ± 15 vs. 65 ± 13 ms; *p* = 0.010) were also significantly prolonged. P-wave morphology was similar between subgroups.

Regarding ventricular conduction, the QRS duration did not differ (99 ± 16 vs. 97 ± 15 ms; *p* = 0.387). The QT interval was longer in the higher risk group (412 ± 38 vs. 395 ± 34 ms; *p* = 0.002), as was QTc (440 ± 32 vs. 425 ± 28 ms; *p* = 0.001). The PR interval showed no significant difference.

Table 5 depicts logistic regression analysis identifying whether PWIs predicted the presence of MAFLD. In unadjusted analyzes, each 1 ms increase in PWdis was associated with 5% higher odds of MAFLD (OR 1.05; 95% CI 1.02–1.08; *p* = 0.001). PWPT-D2 showed the strongest univariate association (OR 1.08; 95% CI 1.05–1.11; *p* < 0.001). PWPT-V1 was also significant in univariate analysis (OR 1.04; 95% CI 1.01–1.07; *p* = 0.004), whereas PWD and PTFV1 did not reach significance (*p* = 0.042 and *p* = 0.085, respectively).

After adjustment for the confounding factors in Model 1, only PWPT-D2 (OR 1.07; 95% CI 1.04–1.10; *p* < 0.001) and PWdis (OR 1.04; 95% CI 1.01–1.07; *p* = 0.009) remained significant predictors. In Model 2, PWPT-D2 (OR 1.06; 95% CI 1.03–1.09; *p* < 0.001) and PWdis (OR 1.03; 95% CI 1.00–1.06; *p* = 0.039) continued to be significant, while PWPT-V1 lost significance (*p* = 0.157). In the fully adjusted Model 3, PWdis (OR 1.03; 95% CI 1.00–1.06; *p* = 0.048) and PWPT-D2 (OR 1.05; 95% CI 1.02–1.08; *p* = 0.002) remained independently associated with MAFLD status; PWPT-V1 did not emerged as statistically significant (*p* = 0.421).

Univariate linear regressions (Table 6) demonstrated that PWdis (B 0.32 ± 0.08; β 0.12; t 4.00; *p* < 0.001) and PWPT-D2 (B 0.45 ± 0.10; β 0.17; t 4.50; *p* < 0.001) were significant predictors of FLI. PWD (*p* = 0.222), PTFV1 (*p* = 0.243), and PWPT-V1 (*p* = 0.468) were not significantly associated in univariate models.

The multivariate model adjusted for the following confounders: age, gender, body mass index, waist circumference, triglycerides, gamma-glutamyl transferase, smoking history, hypertension, diabetes mellitus, left atrial area, and E/e′ lateral ratio.

In the multivariate model (adjusted for BMI, gender, age, waist circumference, triglycerides, GGT, smoking, hypertension, DM, LA area, and E/e′ lateral), PWdis (B 0.27 ± 0.10; β 0.09; t 2.70; *p* = 0.007) and PWPT-D2 (B 0.31 ± 0.13; β 0.12; t 2.38; *p* = 0.018) remained independent predictors of FLI. PWD, PTFV1, and PWPT-V1 were not significant after adjustment.

**Figure 2:**
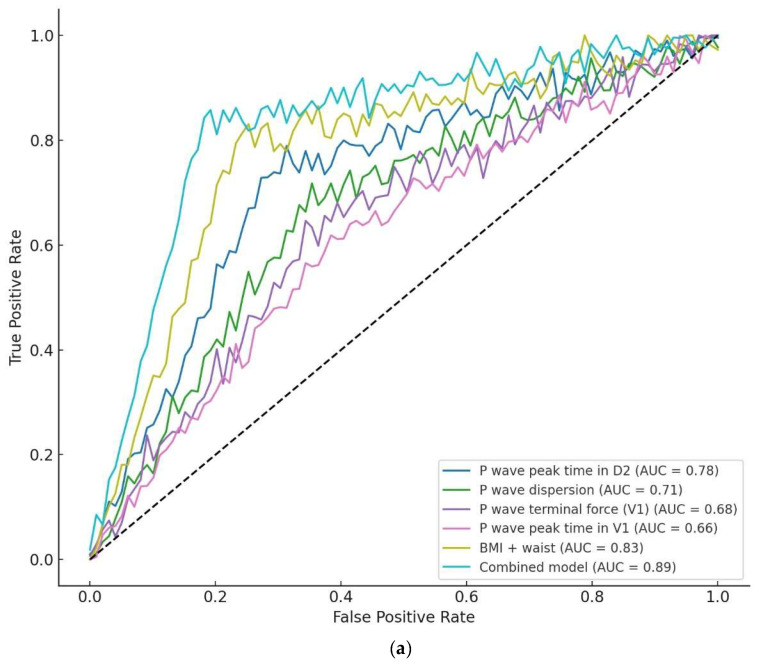

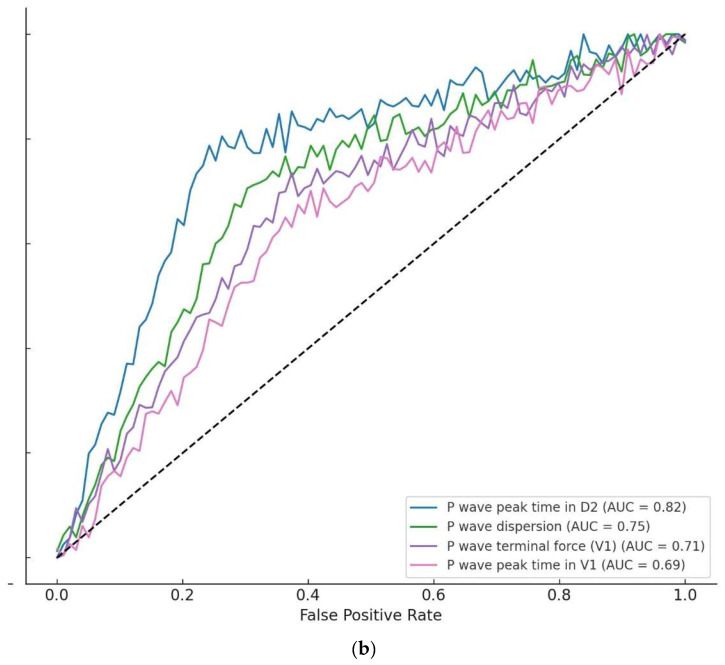
Receiver operating characteristic (ROC) curves comparing the predictive performance of electrocardiographic and combined models for MAFLD detection and fibrosis prediction. (**a**) MAFLD detection (noMAFLD vs. MAFLD). P-wave peak time in D2 (AUC = 0.78; 95% CI 0.72–0.84; optimal cutoff >58 ms; sensitivity 74%; specificity 72%; *p* < 0.001). P-wave dispersion (AUC = 0.71; 95% CI 0.65–0.77; cutoff >50 ms; sensitivity 68%; specificity 66%; *p* < 0.001). P-wave terminal force in V1 (AUC = 0.68; 95% CI 0.61–0.75; cutoff >36 mm·ms; sensitivity 65%; specificity 63%; *p* = 0.002). P-wave peak time in V1 (AUC = 0.66; 95% CI 0.59–0.73; cutoff >64 ms; sensitivity 62%; specificity 61%; *p* = 0.010). Body mass index + waist circumference (AUC = 0.83; 95% CI 0.78–0.88; sensitivity 80%; specificity 76%; *p* < 0.001). The combined electrophysiological + metabolic model (AUC = 0.89; 95% CI 0.85–0.93; sensitivity 84%; specificity 82%; *p* < 0.001). (**b**) Fibrosis prediction among MAFLD patients (NFS < −1.455 vs. ≥ −1.455). P-wave peak time in D2 (AUC = 0.82; 95% CI 0.76–0.88; cutoff >62 ms; sensitivity 78%; specificity 76%; *p* < 0.001). P-wave dispersion (AUC = 0.75; 95% CI 0.68–0.82; cutoff >54 ms; sensitivity 72%; specificity 69%; *p* < 0.001). P-wave terminal force in V1 (AUC = 0.71; 95% CI 0.64–0.78; cutoff >39 mm·ms; sensitivity 68%; specificity 65%; *p* = 0.002). P-wave peak time in V1 (AUC = 0.69; 95% CI 0.62–0.76; cutoff >68 ms; sensitivity 64%; specificity 63%; *p* = 0.008).

The discriminative ability of individual PWIs were studied and combined models for MAFLD detection and fibrosis prediction were interrogated by ROC curve analyzes. For MAFLD versus noMAFLD, PWPT-D2 yielded an AUC of 0.78 (95% CI: 0.72–0.84) at an optimal cutoff >58 ms, corresponding to 74% sensitivity and 72% specificity. PWdis demonstrated an AUC of 0.71 (95% CI: 0.65–0.77) with a cutoff >50 ms (68% sensitivity, 66% specificity), while PWPT-V1 and PWTFV1 exhibited AUCs of 0.66 (95% CI: 0.59–0.73) and 0.68 (95% CI: 0.61–0.75), respectively. By contrast, a combined model incorporating PWPT-D2, BMI, and waist circumference achieved a markedly higher AUC of 0.89 (95% CI: 0.85–0.93), significantly outperforming PWPT-D2 alone (*p* < 0.001 by DeLong’s test), with 84% sensitivity and 82% specificity (Figure 2a).

Among MAFLD patients stratified by fibrosis risk, PWPT-D2 again proved the strongest predictor of a higher fibrosis risk, yielding a cutoff >62 ms (78% sensitivity, 76% specificity) with an AUC of 0.82 (95% CI: 0.76–0.88). While PTFV1 and PWPT-V1 showed a more modest performance (AUCs of 0.71 and 0.69, respectively), the AUC for PWdis was 0.75 (95% CI: 0.68–0.82; cutoff >54 ms, 72% sensitivity, 69% specificity) (Figure 2b).

## 4. Discussion

The most crucial conclusions of the present study are as follows: MAFLD patients had a greater PWdis, PWPT-D2, PWPT-V1, and PTFV1 compared with those without MAFLD. Furthermore, MAFLD patients with a greater fibrosis risk had an also greater PWdis, PWPT-D2, PWPT-V1, and PTFV1 compared with those MAFLD patients with a lower fibrosis risk. PWPT-D2 and PWdis were associated with higher FLI values, increased MAFLD prevalence, and a greater fibrosis risk among MAFLD patients. In ROC analysis, PWPT-D2 achieved the highest AUC among all P-wave indices, indicating a superior discriminative performance. Furthermore, incorporating PWPT-D2 into anthropometric risk models markedly improved MAFLD detection compared with using the P-wave indices alone. Our study is the first to extensively evaluate PWIs across MAFLD patients stratified by fibrosis risk, to the best of our knowledge.

Atrial tachyarrhythmias, specifically AF, are frequently observed in patients with MAFLD, especially among those who have concomitant DM [16]. Targher et al. [24] demonstrated in their longitudinal cohort of 400 subjects with DM that MAFLD independently predicted a higher incidence of AF over a decade, even following adjustment for sex, age, hypertension, and ECG parameters. An analysis including observational data further corroborated the link between MAFLD and an elevated AF risk [7]. Shared pathogenic drivers—such as obesity, systemic inflammation, and oxidative stress—likely underpin atrial remodeling and electrical instability in this population [16]. In addition to AF, other malignant arrhythmias appear more common in MAFLD: one large cohort by Hung et al. [8] revealed that QTc interval prolongation increased in parallel with MAFLD severity. Another study by Naderi et al. [9] on a large population consisting of 4603 MAFLD patients approved this QTc prolongation. Epicardial fat accumulation in MAFLD alters myocardial energy utilization, promoting left ventricular diastolic dysfunction and, consequently, LA enlargement and impaired atrial mechanics [25]. Consistent with this, El Sharkawy et al. observed that MAFLD patients exhibited subclinical impairments in LA conduit and contractile phases, along with abnormal left ventricular diastolic parameters [26]. In our study, these atrial changes may be reflected by a prolonged PWPT-D2 and increased PWdis.

Multiple pathways have been proposed to explain the heightened arrhythmic risk in NAFLD. Increased epicardial and pericardial fat contributes to both systemic and myocardial inflammation by releasing proinflammatory cytokines such as TNF-alpha and IL-6, while simultaneously reducing protective adipokines like adiponectin, thus promoting an environment conducive to arrhythmias [27]. These cytokines have been shown to induce a structural and electrical remodeling of the atrial myocardium by promoting fibrosis and altering gap junction expression and function, particularly connexin-43, thereby disrupting atrial conduction pathways [28]. Such remodeling facilitates heterogeneous impulse propagation and predisposes to atrial arrhythmias, including atrial fibrillation. Moreover, chronic inflammation and oxidative stress in MAFLD exacerbate autonomic dysfunction, further destabilizing cardiac electrophysiology. These mechanistic insights are supported by experimental studies demonstrating that IL-6 mediates gap junction remodeling and arrhythmogenic substrate formation, underscoring the pathophysiological link between hepatic metabolic dysfunction and atrial electrical disturbances [10,28]. Elevated circulating free fatty acids in NAFLD are stored as myocardial triglycerides, precipitating cardiomyocyte apoptosis and lipotoxic damage that can destabilize electrical conduction. Furthermore, NAFLD is associated with impaired cardiac autonomic regulation, which may further predispose to arrhythmias [28]. Insulin resistance and alterations in gut microbiota have also been implicated in promoting both structural remodeling and electrical disturbances within the myocardium [10]. Continued research into these pathophysiological processes is essential to refine risk stratification and optimize therapeutic strategies for NAFLD patients.

Research linking P-wave characteristics to atrial fibrillation has expanded considerably. Consequently, P-wave analysis is increasingly utilized to assess AF risk in patients who might gain from targeted preventive strategies. PWPT is a novel ECG parameter under continuous investigation. While a normal P-wave represents the depolarization of both atria, LA activation dominates the waveform due to its greater myocardial mass. Consequently, the P-wave apex occurs later as the impulse traverses the larger left atrium. Çınar et al. [13] demonstrated in their study that PWPT-D2 was linked with the emergence of AF in acute ischemic stroke patients. Similarly, Karayakali et al. [29] reported an increased PWPT-D2 in implantable cardiac device patients with high atrial rate episodes. Çetinarslan et al. [30] reported in their study that PWPT-D2 but not PWPT-V1 was associated with postoperative AF occurrence in liver transplant patients. PWPT was also found to be associated with deranged left ventricular diastolic functions. Burak et al. [31] showed that PWPT-D2 >60 ms was correlated with an increased LA volume index and escalated LV diastolic dysfunction. They concluded that PWPT-D2 might be utilized as a simple marker of LA mechanics to predict LV diastolic functions. A study by Yıldız et al. [15] reported that an increased LA volume index > 28 mL/m^2^ was associated with a PWPT-D2 >60 ms. In our study, we found a ROC-derived cutoff value of 58 ms for the prediction of MAFLD and 62 ms for the prediction of higher fibrosis risk among MAFLD patients; furthermore, the mean LA area was greater in the MAFLD group compared with the controls. Therefore, our findings seem compatible with the existing literature.

PWdis reflects the nonuniform, fragmented conduction of sinus impulses and has been linked to the development of AF [32]. An increased PWdis was found to be associated with AF recurrence after successful cardioversion [33]. In addition, Çağdaş et al. [14] investigated PWIs including PWPT-D2, PWPT-V1, PWdis, PWTFV1, and PWD, and found that only PWPT-D2 and PWdis were significantly associated with silent cerebral ischemia and future cerebrovascular events. Our findings seem parallel to their findings.

Our current findings build upon these observations by demonstrating that PWPT-D2 and PWdis—noninvasive markers of atrial conduction delay—are significantly prolonged in MAFLD patients relative to non-MAFLD controls, and are further accentuated in those with advanced fibrosis. In ROC analyzes, PWPT-D2 emerged as the single most robust predictor of both MAFLD presence and fibrosis severity, suggesting that electrical remodeling may precede overt atrial arrhythmias. These results complement prior work linking MAFLD to AF and QTc prolongation by highlighting early, subclinical atrial changes detectable on standard ECG. Together, these data underline how significant vigilant cardiac monitoring in MAFLD is, as subtle P-wave abnormalities may herald progression to clinically significant atrial tachyarrhythmias.

This study provides critical evidence linking atrial ECG signals to MAFLD prevalence and fibrosis risk. Our results highlight PWPT-D2 as a noninvasive and hypothesis-generating candidate marker for identifying MAFLD and staging fibrosis, especially when used alongside anthropometric measures. Diagnostic performance improved significantly with a combined model incorporating ECG markers (especially PWPT-D2) alongside metabolic risk indicators (BMI and waist circumference), reaching an AUC of 0.89. This multi-modal approach could enhance MAFLD screening and stratification in primary care settings, proposing a noninvasive and cheap method to specify risky patients for hepatic and cardiovascular complications. Consequently, PWPT-D2 shows promise as a clinical tool for both diagnosing MAFLD and predicting future atrial arrhythmias. Future research should focus on validating a PWPT-D2 threshold in larger, ethnically diverse cohorts with longitudinal follow-up to confirm their predictive value for MAFLD, fibrosis progression, and incident atrial arrhythmias. Integrating these ECG markers into multimodal algorithms—alongside noninvasive fibrosis scores, imaging data, and metabolic biomarkers—could enhance risk stratification, particularly if machine learning methods optimize variable weighting.

## 5. Limitations

Several constraints of this study merit consideration. Firstly, its retrospective design inherently limits causal inferences and may introduce selection bias, as only individuals who underwent routine cardiac evaluation were included. Secondly, hepatic steatosis was defined by the FLI rather than imaging or biopsy; although FLI has been validated as a surrogate for steatosis, it may misclassify some patients and cannot distinguish between simple steatosis and steatohepatitis. Thirdly, fibrosis staging relied on the NFS rather than elastography or histology, potentially underestimating or overestimating the fibrosis severity in certain subgroups. Fourthly, our cohort was drawn from only one tertiary-care hospital, which might limit generalizability to broader outpatient populations or different ethnic groups. Fifthly, although PWPT-D2 and PWdis correlated with MAFLD and fibrosis risk, we did not prospectively track incident atrial arrhythmias (e.g., AF) to confirm their prognostic utility. Finally, unmeasured confounders—such as medication changes over time, dietary patterns, or genetic factors—could have influenced both ECG parameters and liver pathology. Future prospective, multicenter studies incorporating imaging or histologic confirmation of liver disease and longitudinal arrhythmia monitoring are warranted to corroborate our findings and make clearer the temporal relationship between P-wave abnormalities and cardiovascular outcomes in MAFLD, and test our findings in independent external cohorts. Our results only reflect the exploratory nature of our work and do not overstate the immediate clinical readiness of P-wave indices. Without longitudinal follow-up linking P-wave abnormalities to incident arrhythmias or fibrosis progression, these indices cannot yet be recommended for clinical use.

## 6. Conclusions

We demonstrated that PWPT-D2 and PWdis are significantly elevated in MAFLD subjects in comparison with the ones without MAFLD, and these conduction delays are further accentuated in individuals at higher hepatic fibrosis risk. Among all PWIs assessed, PWPT-D2 exhibited the greatest discriminative performance for both MAFLD presence and fibrosis risk and, when combined with anthropometric measures, improved diagnostic accuracy. Our data identify that PWPT-D2 may represent a hypothesis-generating, simple, and noninvasive ECG marker that could facilitate the early identification of MAFLD and fibrosis staging in at-risk populations. The integration of P-wave analysis into routine clinical workflows may enable timely referral for hepatology evaluation and closer cardiac monitoring, ultimately helping to mitigate arrhythmic and cardiovascular complications in MAFLD. Future multicenter prospective investigations are required to corroborate our findings and to determine whether targeted interventions based on P-wave abnormalities can improve liver and cardiac outcomes.

## Figures and Tables

**Table 1:** A comparison of the demographic, laboratory, and echocardiographic features between patients with and without MAFLD.

	noMAFLD (n = 205)	MAFLD (n = 242)	*p*
**Demographics/Clinic**			
Age (years), mean ± SD	56.8 ± 10.9	55.5 ± 10.8	0.180
Female, n (%)	98 (47.8%)	110 (45.5%)	0.622
BMI (kg/m^2^), mean ± SD	24.6 ± 3.2	29.8 ± 4.7	<0.001
Smoker, n (%)	44 (21.5%)	45 (18.6%)	0.450
Diabetes Mellitus, n (%)	25 (12.2%)	75 (31.0%)	<0.001
Hypertension, n (%)	51 (24.9%)	85 (35.1%)	0.018
Chronic Alcohol, n (%)	18 (8.8%)	25 (10.3%)	0.572
Waist Circumference (cm), mean ± SD	91.1 ± 6.4	111.8 ± 12.0	<0.001
**Laboratory**			
Fasting Glucose (mg/dL), median [IQR]	96 [71–177]	100 [77–188]	0.240
Total Cholesterol (mg/dL), mean ± SD	189.5 ± 36.0	193.2 ± 38.3	0.290
Triglycerides (mg/dL), mean ± SD	154.1 ± 70.5	180.5 ± 100.8	0.004
LDL (mg/dL), mean ± SD	110.2 ± 29.5	111.8 ± 36.2	0.620
HDL (mg/dL), mean ± SD	47.5 ± 10.1	46.2 ± 10.0	0.180
AST (U/L), median [IQR]	18 [9–45]	25 [14–82]	<0.001
ALT (U/L), median [IQR]	19 [7–95]	26 [13–152]	<0.001
Bilirubin Total (mg/dL), median [IQR]	0.56 [0.18–1.30]	0.60 [0.24–2.00]	0.430
Bilirubin Direct (mg/dL), median [IQR]	0.13 [0.04–0.42]	0.15 [0.06–0.59]	0.230
ALP (U/L), mean ± SD	72.3 ± 18.5	75.1 ± 21.4	0.125
GGT (U/L), median [IQR]	24 [9–60]	36 [11–92]	0.010
LDH (U/L), mean ± SD	185.1 ± 29.2	191.3 ± 36.5	0.110
INR, mean ± SD	0.99 ± 0.14	1.03 ± 0.11	0.192
Creatinine (mg/dL), mean ± SD	0.87 ± 0.18	0.85 ± 0.19	0.130
GFR (mL/min/1.73 m^2^), mean ± SD	85.5 ± 22.8	86.5 ± 19.3	0.550
Total Protein (g/dL), mean ± SD	75.1 ± 4.4	74.4 ± 4.8	0.120
Albumin (g/dL), mean ± SD	42.2 ± 2.7	42.1 ± 3.0	0.720
Sodium (mmol/L), mean ± SD	139.3 ± 3.2	139.0 ± 3.2	0.310
Potassium (mmol/L), mean ± SD	4.39 ± 0.37	4.38 ± 0.37	0.560
WBC (×10^3^/L), mean ± SD	7.45 ± 1.80	7.42 ± 2.00	0.620
Neutrophil (×10^3^/L), mean ± SD	4.38 ± 1.28	4.35 ± 1.32	0.530
Lymphocyte (×10^3^/L), mean ± SD	2.38 ± 0.70	2.40 ± 0.74	0.800
Monocyte (×10^3^/L), mean ± SD	0.55 ± 0.16	0.54 ± 0.15	0.530
Hemoglobin (g/dL), mean ± SD	14.25 ± 1.48	13.98 ± 1.46	0.065
Platelet (×10^3^/L), mean ± SD	249.0 ± 67.7	258.1 ± 77.5	0.240
Sedimentation, mean ± SD	13.8 ± 11.2	15.5 ± 11.5	0.140
CRP (mg/dL), mean ± SD	3.85 ± 3.70	4.65 ± 5.90	0.160
**Echocardiographic Parameters**			
LVEF (%), mean ± SD	62.1 ± 4.8	61.8 ± 5.0	0.520
Left Atrium Area (cm^2^), median [IQR]	22.8 [18.9–25.7]	28.3 [24.2–31.5]	<0.001
E/e’ lateral, mean ± SD	8.2 ± 2.1	10.5 ± 3.3	<0.001
E/e’ medial, mean ± SD	6.5 ± 1.8	8.9 ± 2.7	<0.001
E/A Ratio, mean ± SD	1.02 ± 0.31	0.98 ± 0.29	0.210
Deceleration Time (ms), median [IQR]	180 [150–210]	190 [160–220]	0.150
LVEDD (mm), mean ± SD	47.2 ± 3.8	48.3 ± 4.2	0.131
IVS Thickness (mm), mean ± SD	9.7 ± 1.2	9.8 ± 1.4	0.294
PWT (mm), mean ± SD	8.9 ± 1.1	9.2 ± 1.3	0.372
**Medications**			
Oral Antidiabetics, n (%)	22 (10.7%)	30 (12.4%)	0.612
Insulin, n (%)	8 (3.9%)	12 (5.0%)	0.652
Statins, n (%)	35 (17.1%)	48 (19.8%)	0.478
Beta Blockers, n (%)	42 (20.5%)	55 (22.7%)	0.642
Calcium Channel Blockers, n (%)	28 (13.7%)	38 (15.7%)	0.589
ACEI/ARBs, n (%)	45 (22.0%)	60 (24.8%)	0.532

LVEF, left ventricular ejection fraction; LVEDD, left ventricular end-diastolic diameter; IVS, interventricular septum; PWT, posterior wall thickness; BMI, body mass index; LDL, low-density lipoprotein; HDL, high-density lipoprotein; AST, aspartate transaminase; ALT, alanine transaminase; ALP, alkaline phosphatase; GGT, gamma-glutamyl transferase; INR, international normalized ratio; GFR, glomerular filtration rate; CRP, C-reactive protein; ACE/ARBs, angiotensin-converting enzyme inhibitors/angiotensin receptor blockers.

**Table 2:** Comprehensive characteristics of MAFLD patients by fibrosis stage.

	MAFLD with Lower Fibrosis Risk (n = 170) (NFS Score < −1.455)	MAFLD with Higher Fibrosis Risk (n = 72) (NFS Score > −1.455)	*p*
NFS score, median [IQR]	−1.9 [−2.4–−1.5]	0.4 [−0.8–1.6]	<0.001
**Demographic/Clinic**			
Age (years), mean ± SD	53.2 ± 10.4	59.7 ± 9.8	<0.001
Female, n (%)	78 (45.9%)	32 (44.4%)	0.842
BMI (kg/m^2^), mean ± SD	29.1 ± 4.5	31.5 ± 4.9	0.002
Waist circumference (cm), mean ± SD	110.2 ± 11.5	115.3 ± 12.8	0.013
Diabetes, n (%)	45 (26.5%)	30 (41.7%)	0.018
Hypertension, n (%)	53 (31.2%)	32 (44.4%)	0.049
Smoker, n (%)	32 (18.8%)	13 (18.1%)	0.892
Chronic alcohol, n (%)	16 (9.4%)	9 (12.5%)	0.452
**Laboratory**			
AST (U/L), median [IQR]	23 [16–38]	42 [28–68]	<0.001
ALT (U/L), median [IQR]	28 [18–44]	48 [32–130]	<0.001
GGT (U/L), median [IQR]	32 [18–76]	58 [35–115]	<0.001
ALP (U/L), mean ± SD	74.3 ± 20.1	77.2 ± 24.3	0.352
Bilirubin total (mg/dL), median [IQR]	0.58 [0.22–1.95]	0.65 [0.28–2.20]	0.128
INR, mean ± SD	1.02 ± 0.10	1.05 ± 0.13	0.064
Fasting glucose (mg/dL), median [IQR]	98 [75–182]	108 [85–210]	0.005
Triglycerides (mg/dL), mean ± SD	175.3 ± 95.2	193.8 ± 112.4	0.032
HDL (mg/dL), mean ± SD	46.5 ± 9.8	45.2 ± 10.5	0.382
LDL (mg/dL), mean ± SD	110.8 ± 35.1	114.2 ± 38.7	0.512
CRP (mg/dL), mean ± SD	4.2 ± 5.1	5.8 ± 7.3	0.048
Platelets (×10^3^/L), mean ± SD	255.1 ± 72.3	235.8 ± 68.9	0.046
**Echocardiography**			
LVEF (%), mean ± SD	62.0 ± 4.9	61.4 ± 5.2	0.410
E/e’ lateral, mean ± SD	9.5 ± 2.6	12.2 ± 3.5	<0.001
E/e’ medial, mean ± SD	7.8 ± 2.3	9.6 ± 3.0	<0.001
Left atrium area (cm^2^), median [IQR]	27.1 [23.5–30.0]	30.5 [26.8–34.0]	<0.001
LVEDD (mm), mean ± SD	48.1 ± 4.0	49.8 ± 4.5	0.008
IVS thickness (mm), mean ± SD	9.8 ± 1.3	10.2 ± 1.5	0.041
PWT (mm), mean ± SD	9.2 ± 1.2	9.7 ± 1.4	0.009
**Medications**			
Oral antidiabetics, n (%)	20 (11.8%)	10 (13.9%)	0.682
Insulin, n (%)	10 (5.9%)	16 (22.2%)	<0.001
Statins, n (%)	32 (18.8%)	21 (29.2%)	0.074
Beta blockers, n (%)	38 (22.4%)	17 (23.6%)	0.832
ACEI/ARBs, n (%)	40 (23.5%)	20 (27.8%)	0.482

LVEF, left ventricular ejection fraction; LVEDD, left ventricular end-diastolic diameter; IVS, interventricular septum; PWT, posterior wall thickness; BMI, body mass index; LDL, low-density lipoprotein; HDL, high-density lipoprotein; AST, aspartate transaminase; ALT, alanine transaminase; ALP, alkaline phosphatase; GGT, gamma-glutamyl transferase; INR, international normalized ratio; CRP, C-reactive protein; ACE/ARBs, angiotensin-converting enzyme inhibitors/angiotensin receptor blockers.

**Table 3:** A comparison of electrocardiographic parameters between patients with and without MAFLD.

	noMAFLD (n = 205)	MAFLD (n = 242)	*p*
Heart rate (bpm)	72 ± 13	75 ± 14	0.089
P-wave duration D2 (ms)	92 ± 13	94 ± 12	0.245
P-wave max (ms)	118 ± 15	121 ± 14	0.189
P-wave min (ms)	72 ± 9	74 ± 10	0.157
P-wave dispersion (ms)	46 ± 11	55 ± 13	0.010
P-wave terminal force V1 (mm·ms)	28 [22–34]	38 [31–46]	0.021
P-wave peak time D2 (ms)	52 ± 10	63 ± 12	0.003
P-wave peak time V1 (ms)	60 ± 13	68 ± 14	0.005
QRS duration (ms)	96 ± 16	98 ± 15	0.351
QT interval (ms)	380 ± 32	400 ± 36	<0.001
QTc (Bazett, ms)	410 ± 25	430 ± 30	<0.001
PR interval (ms)	152 ± 22	155 ± 24	0.287
P-wave morphology in V1			0.901
Negative, n (%)	4 (2.0%)	5 (2.1%)	
Positive, n (%)	56 (27.3%)	68 (28.1%)	
Biphasic (±), n (%)	145 (70.7%)	169 (69.8%)	

**Table 4:** A comparison of electrocardiographic parameters among MAFLD patients according to hepatic fibrosis risk.

	NFS < −1.455 (Lower Fibrosis)	NFS ≥ −1.455 (Higher Fibrosis)	*p*-Value
Heart rate (bpm)	74 ± 13	77 ± 15	0.152
RR interval (ms)	811 ± 148	780 ± 151	0.138
P-wave duration D2 (ms)	92 ± 12	95 ± 13	0.201
P-wave max (ms)	119 ± 14	122 ± 15	0.183
P-wave min (ms)	73 ± 9	75 ± 10	0.241
P-wave dispersion (ms)	52 ± 12	58 ± 14	0.006
P-wave terminal force V1 (mm·ms)	35 [29–42]	42 [35–50]	0.015
P-wave peak time D2 (ms)	60 ± 11	66 ± 13	0.008
P-wave peak time V1 (ms)	65 ± 13	71 ± 15	0.010
P-wave morphology in V1			0.887
Negative, n (%)	3 (1.8%)	2 (2.8%)	
Positive, n (%)	48 (28.2%)	20 (27.8%)	
Biphasic (±), n (%)	119 (70.0%)	50 (69.4%)	
QRS duration (ms)	97 ± 15	99 ± 16	0.387
QT interval (ms)	395 ± 34	412 ± 38	0.002
QTc (Bazett, ms)	425 ± 28	440 ± 32	0.001
PR interval (ms)	154 ± 23	157 ± 25	0.421

**Table 5:** Logistic regression analysis for MAFLD prediction using P-wave indices.

	Unadjusted OR (95% CI) *p*-Value	Model 1 OR (95% CI) *p*-Value		Model 2 OR (95% CI) *p*-Value		Model 3 OR (95% CI) *p*-Value
P-wave duration D2 (ms)	1.02 (1.00–1.04) *p* = 0.042 *	1.01 (0.99–1.03) *p* = 0.198		1.00 (0.98–1.02) *p* = 0.712		0.99 (0.97–1.01) *p* = 0.382
P-wave dispersion (ms)	1.05 (1.02–1.08) *p* = 0.001 **	1.04 (1.01–1.07) *p* = 0.009 **		1.03 (1.00–1.06) *p* = 0.039 *		1.03 (1.00–1.06) *p* = 0.048 *
P-wave terminal force V1 (mm·ms)	1.02 (1.00–1.04) *p* = 0.085	1.01 (0.99–1.03) *p* = 0.324		1.00 (0.98–1.02) *p* = 0.512		0.99 (0.97–1.01) *p* = 0.287
P-wave peak time D2 (ms)	1.08 (1.05–1.11) *p* < 0.001 ***	1.07 (1.04–1.10) *p* < 0.001 ***		1.06 (1.03–1.09) *p* < 0.001 ***		1.05 (1.02–1.08) *p* = 0.002 **
P-wave peak time V1 (ms)	1.04 (1.01–1.07) *p* = 0.004 **	1.03 (1.00–1.06) *p* = 0.023 *		1.02 (0.99–1.05) *p* = 0.157		1.01 (0.98–1.04) *p* = 0.421
**Model Fit Statistics for MAFLD Prediction Models**						
**Fit Metric**	**Model 1** **(Demographics + Obesity)**		**Model 2** **(+ Metabolic Factors)**		**Model 3** **(+ Cardiac Remodeling)**	
Omnibus Test (χ^2^, *p*-value)	38.6, *p* < 0.001 ***		52.3, *p* < 0.001 ***		61.8, *p* < 0.001 ***	
Nagelkerke R^2^	0.28		0.36		0.41	
Hosmer–Lemeshow Test (χ^2^, *p*-value)	6.24, *p* = 0.398		5.87, *p* = 0.438		4.92, *p* = 0.553	
Classification Accuracy (%)	72.3%		75.1%		77.6%	
AUC (95% CI)	0.75 (0.70–0.80)		0.80 (0.76–0.84)		0.83 (0.79–0.87)	
AIC	538.2		522.7		513.9	
BIC	572.4		576.1		584.8	

* *p* < 0.05, ** *p* < 0.01, *** *p* < 0.001. Model 1: adjusted for age, sex, BMI, and waist circumference. Model 2: adjusted for Model 1 + metabolic factors. Model 3: adjusted for Model 2 + echocardiographic remodeling. The Omnibus test assesses overall model significance. Hosmer–Lemeshow *p* > 0.05 indicates good fit. AIC: Akaike Information Criterion; BIC: Bayesian Information Criterion. OR: odds ratio.

**Table 6:** Univariate and multivariate linear regression analysis of P-wave parameters predicting FLI.

	Univariate				Multivariate			
	B (SE)	β	t	*p*-Value	B (SE)	β	t	*p*-Value
P-wave dispersion (ms)	0.32 (0.08)	0.12	4.00	<0.001	0.27 (0.10)	0.09	2.70	0.007
P-wave duration in D2 (ms)	0.11 (0.09)	0.04	1.22	0.222	0.05 (0.11)	0.02	0.45	0.650
P-wave terminal force in V1 (mm·ms)	−0.14 (0.12)	−0.05	−1.17	0.243	−0.12 (0.15)	−0.04	−0.80	0.425
P-wave peak time in D2 (ms)	0.45 (0.10)	0.17	4.50	<0.001	0.31 (0.13)	0.12	2.38	0.018
P-wave peak time in V1 (ms)	0.08 (0.11)	0.03	0.73	0.468	0.03 (0.14)	0.01	0.21	0.832

Multivariate Model Fit Statistics: R^2^ = 0.28; Adjusted R^2^ = 0.26; F (15, 431) = 11.57, *p* < 0.001; Durbin–Watson = 1.94.

## Data Availability

Data are available from the corresponding author upon reasonable request.
